# Comparing the Efficacy and Safety of Intra-articular Injection Treatments for Hip Osteoarthritis: A Systematic Review and Network Meta-analysis

**DOI:** 10.1177/23259671261419519

**Published:** 2026-04-13

**Authors:** Chunlin Liu, Jiawen Deng, Aazad Abbas, Darius Luke Lameire, Hassaan Abdel Khalik, Daniel Whelan, Tim Dwyer, Jaskarndip Chahal

**Affiliations:** † University of Toronto Faculty of Medicine, Toronto, Canada; ‡ McMaster University Division of Orthopaedic Surgery, Hamilton, Canada; § University of Toronto Orthopaedic Sports Medicine, Toronto, Canada; ‖ Women's College Hospital, Division of Orthopaedic Surgery, Toronto, Canada; ¶ St Michael's Hospital, Division of Orthopaedic Surgery, Toronto, Canada; # Mount Sinai Hospital, Division of Orthopaedic Surgery, Toronto, Canada; Investigation performed at University of Toronto, Toronto, Canada

**Keywords:** hip osteoarthritis, intra-articular injection, network meta-analysis

## Abstract

**Background:**

Hip osteoarthritis is a debilitating condition that leads to progressive joint pain and stiffness. While total hip arthroplasty provides definitive treatment, intra-articular injections offer a less invasive alternative for patients. Several injection options are available, including corticosteroids (CS), hyaluronic acid (HA), and platelet-rich plasma (PRP). Previous reviews and network meta-analyses have compared the short-term efficacy of these injections, but it remains unclear if a particular injection provides superior symptom relief for up to 12 months.

**Purpose:**

To provide an updated summary of the current hip intra-articular injection literature and compare the efficacy of all injection types at 3 months, 6 months, and 12 months.

**Study Design:**

Systematic review; Level of evidence, 1.

**Methods:**

Four databases were queried: Web of Science, Embase, MEDLINE, and Cochrane Central Register of Controlled Trials. The primary outcome measures were the Western Ontario and McMaster Universities Osteoarthritis Index Total Score (WOMAC–Total) and the visual analog scale (VAS) at 3, 6, and 12 months. The Cochrane risk-of-bias tool was used to assess study quality. Treatment effects were expressed as mean differences for the WOMAC–Total and standardized mean differences for the VAS.

**Results:**

A total of 14 studies were included in the final analysis with 1254 participants. Eight unique intra-articular injection types were identified: CS, HA of varying molecular weights (low, high, and ultra-high), PRP, CS + high molecular weight HA, PRP + HA, and standard of care/placebo (SOC/PBO) group. When compared with SOC/PBO, no statistically significant differences in WOMAC–Total and VAS outcomes were observed between any injections at 3, 6, or 12 months.

**Conclusion:**

There were no statistically significant differences in WOMAC–Total and VAS outcomes at any time point between all injection types to baseline. Future studies should compare the long-term efficacy of various intra-articular injections with a control and examine the efficacy of combined injections.

**Registration:**

CRD42024574937 (PROSPERO identifier).

Hip osteoarthritis (OA) is a debilitating condition that causes pain and stiffness, which can substantially affect patients’ physical function, mental health, and overall quality of life.^29,46^ Many patients suffer from hip OA, as it is estimated to affect 7.95% of the adult population in North America and 12.59% in Europe.^18^ While severe hip OA can be definitively treated with total hip arthroplasty, the procedure may not be suitable for all patients based on the associated risks of the surgery and patients’ own preferences. An alternative to total hip arthroplasty is intra-articular injections, which provide temporary symptom alleviation for patients. Several injection options are available on the market, including corticosteroids (CS), hyaluronic acid (HA), and platelet-rich plasma (PRP).^19,21^ Different types of intra-articular injections act in different ways to relieve hip OA symptoms. For example, CS inhibits the activity of proinflammatory cytokines and helps reduce inflammation by attenuating the immune system.^31^ PRP is derived from autologous blood and contains a high concentration of platelets, growth factors (eg, vascular endothelial growth factor and platelet-derived growth factor), and immunomodulatory cytokines (eg, interleukin 1–beta, IL-1Ra).^52^ It has been suggested that immunomodulatory cytokines reduce inflammation in the joint space, ultimately alleviating hip OA symptoms.^1^ Last, HA alleviates OA by providing chondroprotective, anti-inflammatory, and lubrication effects in the joint space.^17,23^ Given the diverse intra-articular injection options, it can be challenging for clinicians and patients to choose the best option. Hence, it is clinically important to determine the relative efficacy of injections. Elucidating the relative efficacy of these injections will improve outcomes, guide protocols, and optimize the use of health care resources.

Prior reviews and network meta-analyses (NMAs) offer important contributions to the field, but existing results have been based on short-term follow-up durations (eg, up to 6 months for Gazendam et al^21^) or outdated database searches (eg, Zhao et al^51^). Zhao et al also did not differentiate between different formulations of HA, such as high molecular weight (HMW-HA) versus low molecular weight (LMW-HA), and some evidence suggests they may vary in efficacy.^7,37,49,50^ Results from existing NMAs are also inconsistent. Gazendam et al^21^ is the most recent synthesis of the literature and compared the efficacy of HA, CS, and PRP with up to 6 months of follow-up. The authors reported no difference in symptom alleviation between HA, CS, PRP, and PRP + HA compared with controls. Zhao et al compared HA, PRP, CS, and PRP + HA with controls. They reported significant advantages of HA and CS in reducing pain at 3 months compared with controls (saline or local anesthetic injections). The conflicting results in the literature leave the comparative efficacy of available injections unclear.

The current study updates prior work by extending follow-up to 12 months, incorporating new randomized controlled trials (RCTs) published since 2021, and differentiating molecular weight formulations of HA. Compared with a conventional meta-analysis, which only synthesizes outcomes from direct comparisons, NMA facilitates indirect comparisons among treatments within the same network.^20^ These indirect comparisons are advantageous in the presence of literature gaps or limited sample sizes.^11^ The current NMA will evaluate the efficacy of intra-articular injections for managing hip OA at 3, 6, and 12 months.

## Methods

This systematic review and NMA was prospectively registered on PROSPERO. We adhered to The PRISMA (Preferred Reporting Items for Systematic Reviews and Meta-Analyses) Extension Statement for Reporting of Systematic Reviews Incorporating Network Meta-analyses of Health Care Interventions (see complete checklist in Appendix Table A1).^25^

### Study Identification

Four databases were systematically searched for relevant studies from inception to November 20, 2023: Web of Science (Core Collection), Embase, MEDLINE, and Cochrane Central Register of Controlled Trials (CENTRAL). No language or date limits were applied. The search strategy included key terms covering hip OA, injection options, and delivery routes. The complete search strategy is available in Supplemental Material Tables S2 to S5 (available separately). The reference section of previous reviews was also searched for relevant articles.

Inclusion criteria for this review included RCTs investigating adult patients (≥18 years of age) with a diagnosis of hip OA. To be eligible, studies were required to assess the use of intra-articular injections, have a minimum follow-up period of ≥3 months, and report the mean and standard deviation of the Western Ontario and McMaster Universities Osteoarthritis Index Total Score (WOMAC–Total) and the visual analog scale (VAS). The exclusion criteria included studies not available in English, unpublished studies, and cadaveric studies. Additionally, we excluded crossover RCTs, pseudo-randomized RCTs, case studies, case series, retrospective observational studies, and any trial that did not report the mean and standard deviation for WOMAC–Total and VAS.

Two reviewers (C.L. and J.D.) independently screened the articles in duplicate. Eligible studies compared any intra-articular injection therapy with another injection. Studies deemed eligible by both reviewers then underwent subsequent independent full-text screening. Disagreements between the 2 reviewers were resolved through discussion, with 2 authors (D.L.L. and A.A.) providing additional consultation if needed.

### Data Extraction

Two reviewers (C.L. and J.D.) performed data extraction independently and in duplicate using a standardized data extraction form established a priori. Discrepancies during extraction were resolved through discussions between the 2 reviewers. From each included article, the following information was extracted: publication year, number of participants, number of hip joints, mean age, mean body mass index, adverse events (AEs), severe adverse events (SAE), and the Kellgren-Lawrence (KL) grade. We extracted the WOMAC–Total score and the VAS score at baseline, 3 months, 6 months, and 12 months.

### Risk of Bias

The revised Cochrane risk-of-bias tool for randomized trials (RoB2) was used to evaluate the risk of bias for all articles.^45^ The 2 reviewers independently assessed the risk of bias for all articles, and conflicts were resolved with consultation with 2 other authors of the team (D.L.L. and A.A.).

### Statistical Analysis

All statistical analyses were completed on R Statistical Software (Version 1.4.1717) using the netmeta package.^3,36^ For direct comparisons, pairwise meta-analyses were conducted to estimate pooled mean differences and 95% CIs for WOMAC–Total outcomes. For VAS, we calculated the standardized mean differences because of heterogeneity in scale formats across studies. For example, some studies used a 0 to 10 scale (eg, Di Sante et al^16^), while others used a 0 to 100 scale (eg, Rezende et al^38^).

Statistical significance was determined based on the 95% CIs. A comparison was considered statistically significant if the confidence interval did not include the null value. A random-effects model was used to account for potential heterogeneity across studies, with between-study variance (τ^2^) estimated using the DerSimonian-Laird method.^15^ Heterogeneity was further quantified using the *I*^2^ statistic. An *I*^2^ value of 0% indicated no observed heterogeneity, while higher values reflected increasing inconsistency among study results.^24^ In contrast to traditional direct comparisons, NMA evaluated both direct and indirect evidence across a network of treatments. Probability scores (P-scores) were derived from networks and were used to compare the relative efficacy between injections. P-scores are frequentist analogs to the Surface Under the Cumulative Ranking (SUCRA) scores in Bayesian NMAs.^40^ P-scores are derived from point estimates and standard errors of treatment effects. Similar to SUCRA scores, P-scores measure the mean extent of certainty that one injection type is better than another. Unlike SUCRA, P-scores do not reflect Bayesian probability-based ranking and do not require resampling methods.^40^ P-scores range from 0 to 1. A P-score close to 1 indicates that a particular injection is likely the most effective compared with all other injections. In contrast, a P-score of 0 indicates that the treatment is likely the least effective.

A network plot was created to illustrate the relationship between injections among included studies. The size of the nodes was proportional to the number of studies evaluating each injection, and the thickness of the edges represented the relative number of direct connections between the connected nodes. Isolated nodes indicated a lack of direct comparison, which could limit the precision of indirect estimates.

## Results

**Figure 1.**
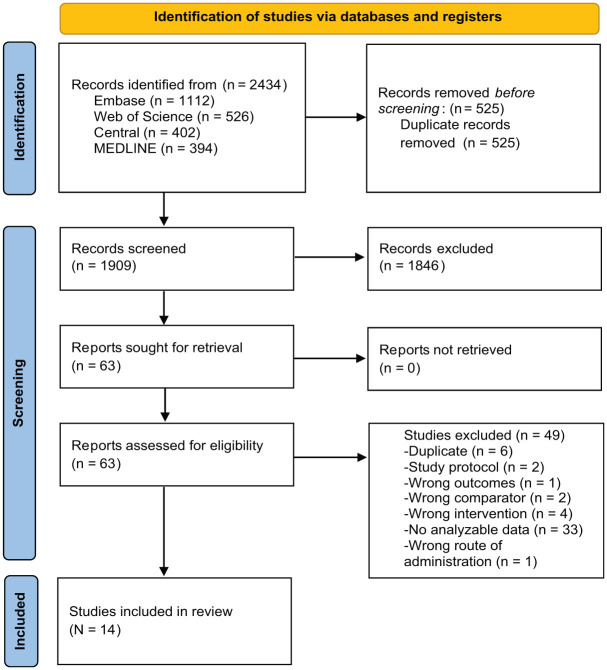
PRISMA (Preferred Reporting Items for Systematic Reviews and Meta-Analyses) flow diagram. The PRISMA flow diagram is adapted from the template outlined in Page et al.^34^

After removing duplicates, a total of 1909 studies were identified. After title and abstract review, 63 studies progressed to full-text review and were assessed for eligibility. Ultimately, 14 studies met the inclusion criteria and were included in the final analysis (Figure 1).

### Study Characteristics

Across all injections, the 14 included studies covered a total of 1254 participants. Table 1 summarizes all included articles. Eight different intra-articular injections were included in the current NMA: (1) CS, (2) ultra–high molecular weight hyaluronic acid (UHMW-HA), (3) HMW-HA, (4) LMW-HA, and (5) PRP. Additionally, combination therapies including (6) CS plus HMW-HA (CS + HMW-HA), (7) PRP plus HMW-HA (PRP + HMW-HA) and standard of care/placebo (SOC/PBO) were included. The (SOC/PBO) group served as a comparator when possible, and included participants who received saline or local anesthetic injections (eg, Migliore et al^32^ used mepivacaine, Richette et al^39^ used saline water). Some studies included a noninjection arm. For example, Paskins et al^35^ had a noninjection arm, which educated patients on noninjection ways to alleviate symptoms, such as exercise and weight loss. These noninjection groups were excluded from the final analysis. The network diagram for WOMAC–Total and VAS at 3 months, 6 months, and 12 months can be found in the Supplemental Material (Figures S1 and S2, available separately).

**Table 1:** Summary of Participant Demographics*^*a*^*

Study	Injections	Total Participants	Total Hip Joints	KL 1 Hips	KL 2 Hips	KL 3 Hips	KL 4 Hips	Age, Mean (SD)	BMI, Mean (SD)
Battaglia 2013^4^	HMW-HA	50	50	NR	23	23	4	56 (12)	27 (4)
	PRP	50	50	NR	16	21	13	51 (12)	26 (5)
Clementi 2018^10^	UHMW-HA	23	23	NR	NR	23	NR	65.9 (10.02)	27.2 (2.38)
	HMW-HA	27	27	NR	NR	27	NR	67.4 (10.3)	26.2 (5.15)
Dallari 2016^13^	HMW-HA	36	37	10	5	12	10	NR	NR
	PRP	44	47	14	10	10	13	NR	NR
	PRP+HMW-HA	31	33	8	8	14	3	NR	NR
Rezende 2020^38^	CS	19	28	NR	18	12	NR	60.1 (12.8)	29.6 (4.6)
	CS+HMW-HA	63	104	NR	65	39	NR	62.5 (12.34)	28.52 (5.89)
Di Sante 2016^16^	HMW-HA	22	22	NR	7	15	NR	73.62 (7.87)	NR
	PRP	21	21	NR	5	16	NR	71.37 (6.03)	NR
Kraeutler 2021^27^	LMW-HA	13	14	NR	2	12	NR	53.6 (7.6)	23.5 (2.0)
	PRP	18	19	NR	7	12	NR	53.3 (8.4)	23.7 (2.1)
Migliore 2009^32^	Local anesthetic	20	27	NR	3	15	2	67 (7.2)	24.8 (4.1)
	HMW-HA	22	34	NR	1	21	NR	68 (10.3)	25.6 (2.2)
Nouri 2022^33^	HMW-HA	29	29	NR	16	13	NR	60.93 (4.54)	27.62 (2.25)
	PRP	32	32	NR	16	15	NR	58.22 (5.1)	27.72 (2.11)
	PRP+HMW-HA	31	31	NR	17	14	NR	60.29 (4.83)	27.94 (2.8)
Paskins 2022^35^	Local anesthetic	66	85	NR	NR	NR	NR	62.3 (9.8)	28.4 (4.9)
	Local anesthetic+ CS	66	80	NR	NR	NR	NR	62.5 (9.3)	29.5 (5.6)
Richette 2009^39^	Saline	43	43	NR	4	39	NR	59.5 (12.6)	26.4 (4.2)
	HMW-HA	42	42	NR	7	35	NR	60.8 (10.2)	26.7 (4.2)
Setaro 2020^41^	HMW-HA	32	32	NR	NR	32	NR	62.5 (11.4)	26.2 (3.7)
	LMW-HA	32	32	NR	NR	32	NR	65.4 (10.7)	27.9 (2.8)
Spitzer 2010^44^	HMW-HA	156	174	NR	57	99	NR	59 (12)	29.3 (5.5)
	CS	156	169	NR	66	90	NR	59 (11)	29.4 (6.0)
Tikiz 2005^47^	HMW-HA	18	24	1	7	16	NR	60.4 (9.6)	29.8 (3.9)
	LMW-HA	25	32	2	10	29	NR	58.8 (9.8)	28.7 (4.3)
Villanova-Lopez 2020^48^	HMW-HA	36	36	13	19	4		61.1 (12.3)	28.4 (4.5)
	PRP	38	38	14	18	6		61.2 (9.72)	28.6 (4.2)

a Levels of hip osteoarthritis severity are reported as KL grade. Overall, HA was the most studied treatment. Note that in Rezende et al,^38^ the 4-mL hylan G-F20 arm showed an inconsistency in the KL grade reported: 36 hips were included, but only 24 KL 2 and 10 KL 3 hips were accounted for. Villanova-Lopez^48^ combined KL 3 and 4. We reported the data the authors presented. BMI, body mass index; CS, corticosteroids; HA, hyaluronic acid; HMW, high molecular weight; KL, Kellgren-Lawrence; LMW, low molecular weight; NR, not reported by the authors or not applicable to the study; PRP, platelet-rich plasma; UHMW, ultra–high molecular weight.

### Risk of Bias

**Figure 2.**
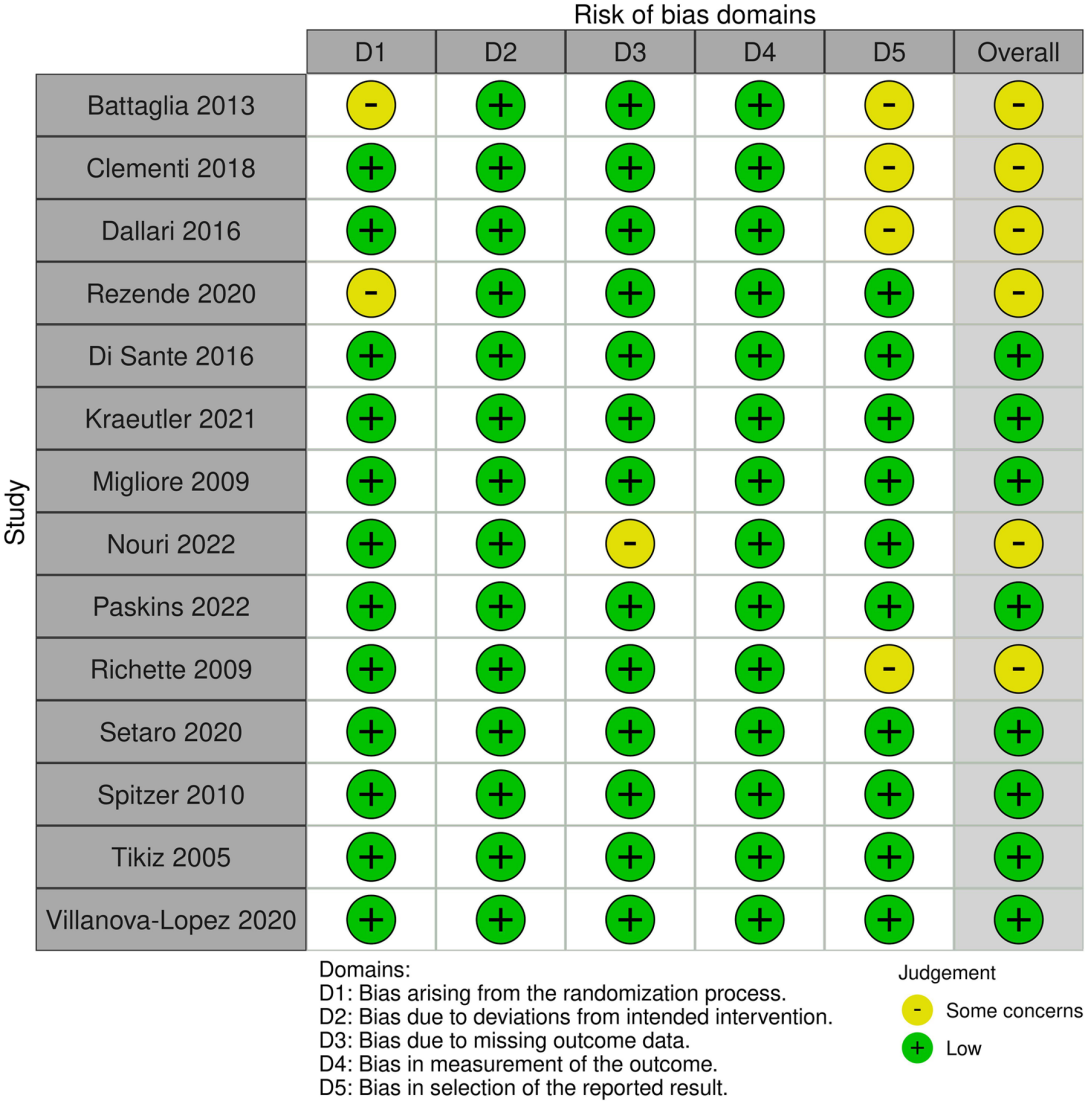
Summary plot for risk of bias. Six studies had some concerns for risk of bias. Bias arose from domain 1 (bias arising from the randomization process), domain 3 (bias due to missing outcome data), and domain 5 (bias in selection of the reported result).

Based on the RoB2 assessment, 8 studies were rated as having a low risk of bias, and 6 studies were rated as having some concerns regarding risk of bias (Figure 2). Most of the bias arose from the randomization process, missing data, and reported results.

### WOMAC–Total

**Figure 3.**
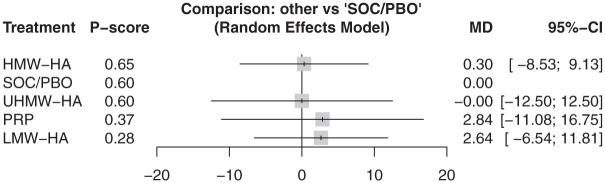
In WOMAC–Total at 3 months, there was no statistically significant difference between all injections and SOC/PBO. HMW-HA ranked highest on the *P* score. HA, hyaluronic acid; HMW, high molecular weight; LMW, low molecular weight; MD, mean difference; PRP, platelet-rich plasma; UHMW, ultra–high molecular weight; SOC/PBO, standard of care/placebo; WOMAC, Western Ontario and McMaster Universities Osteoarthritis Index.

**Figure 4.**
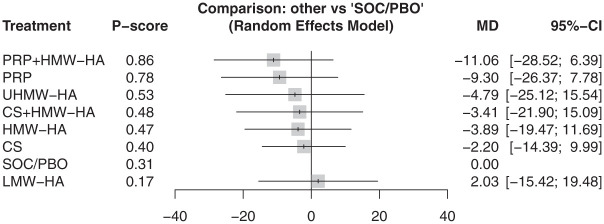
In WOMAC–Total at 6 months, there was no statistically significant difference between all injections and SOC/PBO. PRP+HMW-HA ranked highest on the *P* score. CS, corticosteroids; HA, hyaluronic acid; HMW, high molecular weight; LMW, low molecular weight; MD, mean difference; PRP, platelet-rich plasma; UHMW, ultra–high molecular weight; SOC/PBO, standard of care/placebo; WOMAC, Western Ontario and McMaster Universities Osteoarthritis Index.

**Figure 5.**
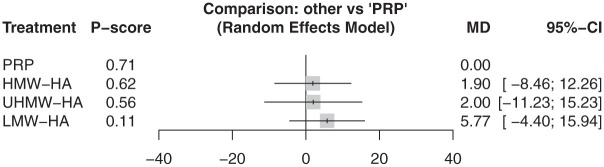
In WOMAC–Total at 12 months, no studies reported results for a standard of care/placebo, so different injection options were compared against PRP. There was no statistically significant difference between all injections against PRP, and PRP ranked highest on the *P* score. HA, hyaluronic acid; HMW, high molecular weight; LMW, low molecular weight; MD, mean difference; PRP, platelet-rich plasma; UHMW, ultra–high molecular weight; WOMAC, Western Ontario and McMaster Universities Osteoarthritis Index.

At 3 months, 6 articles reported the WOMAC–Total score for 5 injections involving 305 participants.^10,27,38,39,41,47^ Results from these 6 articles showed no difference in WOMAC–Total between all injections and SOC/PBO (Figure 3) (τ^2^ = 0.0; *I*^2^ = 0.0%). At 6 months, 9 articles reported 8 injections, covering 941 participants.^**^ These 9 studies also indicated no difference in WOMAC–Total score between all injection types and placebo (Figure 4) (τ^2^ = 24.42; *I*^2^ = 59% [95% CI, 0.0%-83.4%]). At 12 months, 5 articles reported the WOMAC–Total score for 4 injections involving 301 participants.^10,27,38,41,48^ Since no articles reported WOMAC–Total outcomes with SOC/PBO, PRP was set as the baseline, and there was no significant difference between other injections with PRP (Figure 5) (τ^2^ = 0.0; *I*^2^ = 0.0%).

Based on P-score rankings, HMW-HA was the highest-ranking injection at 3 months (P-score, .65). At 6 months, PRP + HMW-HA was the highest-ranking injection (P-score, .86). At 12 months, PRP was the highest-ranking injection (P-score, .71).

### Visual Analog Scale

**Figure 6.**
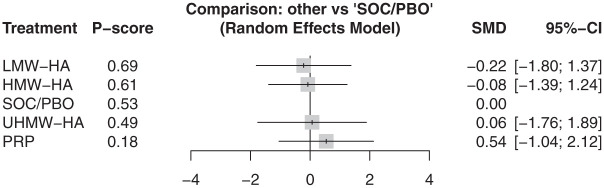
In the visual analog scale at 3 months, there was no statistically significant difference between all injections and SOC/PBO. LMW-HA ranked highest on the *P* score. HA, hyaluronic acid; HMW, high molecular weight; LMW, low molecular weight; SMD, standardized mean difference; PRP, platelet-rich plasma; SOC/PBO, standard of care/placebo; UHMW, ultra–high molecular weight.

**Figure 7.**
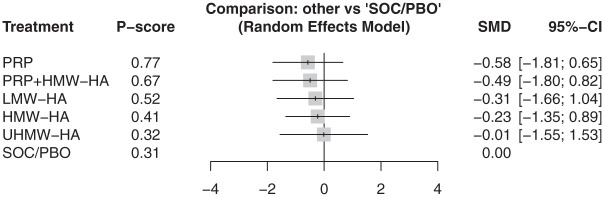
In the visual analog scale at 6 months, there was no statistically significant difference between all injections and SOC/PBO. PRP ranked highest on the *P* score. HA, hyaluronic acid; HMW, high molecular weight; LMW, low molecular weight; SMD, standardized mean difference; PRP, platelet-rich plasma; SOC/PBO, standard of care/placebo; UHMW, ultra–high molecular weight.

**Figure 8.**
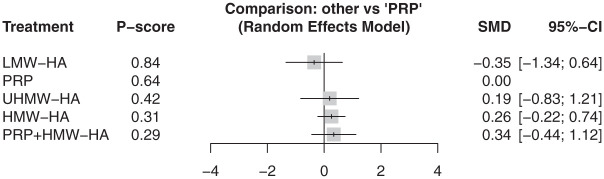
In the visual analog scale at 12 months, no studies reported results for SOC/PBO, so different injection options were compared against PRP. There was no statistically significant difference between all treatments against PRP, and LMW-HA ranked highest on the *P* score. HA, hyaluronic acid; HMW, high molecular weight; LMW, low molecular weight; SMD, standardized mean difference; PRP, platelet-rich plasma; SOC/PBO, standard of care/placebo; UHMW, ultra–high molecular weight.

At 3 months, 6 articles reported pain outcomes covering 381 participants.^4,10,32,38,41,47^ There was no difference between 4 different types of injection treatments against SOC/PBO (Figure 6) (τ^2^ = 0.24; *I*^2^ = 74.2% [95% CI, 28.0%-90.8%]). At 6 months, 7 articles reported 7 injections, involving 497 participants, with no difference across all injection types to SOC/PBO (Figure 7) (τ^2^ = 0.18; *I*^2^ = 74.1% [95% CI, 44.7%-87.9%]).^4,32,33,38,41,47,48^ Finally, at 12 months, 6 articles reported VAS, comparing 5 different injections and including 481 participants.^4,10,13,38,41,48^ However, no article reported outcomes for SOC/PBO at 12 months, so PRP was used as the baseline. All injections had no significant difference compared with PRP at 12 months (Figure 8) (τ^2^ = 0.16; *I*^2^ = 73.6% [95% CI, 26.1%-90.6%]).

At 6 months, PRP was likely the highest-ranking injection (P-score, .77). At 12 months, LMW-HA was the highest-ranking injection (P-score, .84)

### Safety Profile

Nine studies reported AEs related to the injection (Table 2).^††^ The most commonly reported AE was injection-site pain, which was reported across 7 studies affecting 38 participants.^4,13,32,35,39,44,47^ Three studies reported 4 SAEs.^35,39,44^ The SAEs included 1 case of rapidly destructive OA requiring hip replacement, 1 case of subacute bacterial endocarditis, 1 case of spontaneous abortion, and 1 case of choroidal dystrophy (Table 3).

**Table 2:** Adverse Reactions*^*a*^*

Study	Treatment Arm	AE Reported	n/N (%)
Battaglia 2013^4^	HMA-HA	Injection-site pain	10/50 (20.0)
	PRP	Injection-site pain	6/50 (12.0)
Dallari 2016^13^	PRP + HMW-HA	Injection-site pain	13/31 (41.9)
Rezende 2020^38^	CS	Rapid progression to KL grade 4	1/19 (5.3)
	CS+HMW-HA	Rapid progression to KL grade 4	4/63 (6.5)
Migliore 2009^32^	Local anesthetic	Injection-site pain	1/20 (5.0)
	HMW-HA	Hip pain	1/22 (4.5)
Paskins 2022^35^	Local anesthetic	Whitening of skin	2/66 (3.0)
		Thinning of skin	2/66 (3.0)
		Bruising	16/66 (24.2)
		Injection-site pain	7/66 (10.6)
		Other skin problems	2/66 (3.0)
		Infection	1/66 (1.5)
		Anxiety	1/66 (1.5)
	Local anesthetic + CS	Whitening of skin	4/66 (6.1)
		Thinning of skin	2/66 (3.0)
		Bruising	15/66 (25.7)
		Injection-site pain	1/66 (1.5)
		Flushing	4/66 (6.1)
		Menorrhagia	1/66 (1.5)
		Infection	1/66 (1.5)
		Anxiety	1/66 (1.5)
Richette 2009^39^	Saline	Injection-site pain	1/43 (2.3)
		Exacerbation of hip pain	1/43 (2.3)
	HMW-HA	Injection-site pain	3/42 (7.1)
		Pruritus	1/42 (2.4)
		Hematoma	1/42 (2.4)
Spitzer 2010^44^	HMW-HA	Arthralgia	10/156 (6.4)
		Stiffness	3/156 (1.9)
		Asthenia	1/156 (0.6)
		Groin pain	1/156 (0.6)
		Injection-site pain	1/156 (0.6)
	CS	Arthralgia	7/156 (4.5)
		Groin pain	1/156 (0.6)
		Dizziness	1/156 (0.6)
		Feeling hot	1/156 (0.6)
		Hypoesthesia	1/156 (0.6)
		Injection-site pain	1/156 (0.6)
		Muscle spasms	1/156 (0.6)
		Pain in extremity	2/156 (1.2)
		Paraesthesia	1/156 (0.6)
Tikiz 2005^† 47^	LMW-HA	Injection-site pain/swelling	3/32 (9.4)
	HMW-HA	Injection-site pain/swelling	3/24 (12.5)

a AE, adverse event; n, number of participants who reported the specified AE; N, total number of participants in that arm; CS, corticosteroids; HA, hyaluronic acid; HMW, high molecular weight; KL, Kellgren-Lawrence; LMW, low molecular weight; PRP, platelet-rich plasma.

† For Tikiz 2005, n is the number of hips, and N is the total number of hips.

**Table 3:** Severe Adverse Reactions*^*a*^*

Study	Treatment Arm	AE Reported	n/N (%)
Richette 2009^39^	HMW-HA	Rapidly destructive OA requiring hip replacement	1/42 (2.4)
Paskins 2022^35^	Local anesthetic + CS	Subacute bacterial endocarditis	1/66 (1.5)
Spitzer 2010^44^	HMW-HA	Spontaneous abortion	1/156 (2.4)
	CS	Choroidal dystrophy	1/156 (0.6)

a AE, adverse effect; CS, corticosteroids; HA, hyaluronic acide; HMW, high molecular weight; OA, osteoarthritis.

## Discussion

This study provides an updated review and comparison of intra-articular injections for hip OA. The current NMA included 8 different types of intra-articular injections for hip OA: CS, PRP, LMW-HA, HMW-HA, UHMW-HA, PRP + HMW-HA, CS + HMW-HA, and SOC/PBO. The efficacy of these injections was assessed on WOMAC–Total and VAS. We found no statistically significant differences between all injections at 3 months, 6 months, and 12 months on WOMAC–Total and VAS.

The NMA-derived P-scores suggest that some injections may be superior at specific time points, even though direct comparisons revealed no statistically significant differences between them. It is important to acknowledge that P-scores do not directly provide a measure of statistical significance. Instead, P-scores rank injections based on their probability of being superior, considering the point estimates and standard errors across the network.^40^ P-scores should always be interpreted in the context of confidence intervals. While an injection with the highest P-score may appear superior, a confidence interval that includes zero indicates that the difference compared with SOC/PBO is statistically insignificant and the evidence may be inconclusive.^8^

The current NMA shows no statistically significant difference between UHMW-HA, HMW-HA, and LMW-HA. The current evidence adds to the existing literature, which has been inconsistent regarding whether molecular weights affect efficacy. Some evidence suggests that HMW-HA is better than LMW-HA for managing OA symptoms.^37,49,50^ Others have argued that they are comparable.^2,12,22,28,42^ While we report variations in P-score rankings between UHMW-HA, HMW-HA, and LMW-HA, the P-scores alone are insufficient to conclude if one HA formulation is better than another. The rapid degradation of materials in the joint space could account for the lack of significant long-term differences between HA formulations and across all studied injections. For instance, the terminal half-life of corticosteroids in the joint space is less than a week.^14^ Their relatively short half-life could contribute to their short period of efficacy. It has been suggested that HA with a higher molecular weight is better at resisting degradation in the joint space, but results of our NMA do not support this theory.^7,17,30^ The lack of significant long-term differences across all injections reflects the complex pathophysiology of hip OA, and no single treatment can address all aspects of the disease process.

Results from the current NMA have important implications for both clinical practice and future research. Our results show that no injection is superior. Practically, this means clinicians do not need to favor one type over another based solely on efficacy. Instead, factors such as patient preference, cost, and availability can play a larger role in the shared decision-making process. For patients, knowing that all injections perform similarly could have important implications for the cost of care, as some health care systems or insurance plans may only cover certain injections but not others.^5^ Our review of the literature also reveals a gap: no studies have compared an injection with a SOC/PBO group at 12 months of follow-up. This gap makes it difficult to infer if any injection provides benefits beyond SOC/PBO in the long term.

Another gap in the literature is the limited evidence on combination therapies, such as PRP + HMW-HA and CS + HMW-HA. Only 3 studies in our review used a combination therapy. Theoretically, combination therapies provide better symptom alleviation by combining the complementary benefits of different injections. For example, PRP offers regenerative and immunomodulatory effects, while HA contributes mechanical and anti-inflammatory benefits.^9,26^ Although our findings indicate no statistically significant advantage for combination therapies, the lack of significant differences may be due to the limited evidence in the literature and the small sample sizes of the 3 included studies. Meta-analyses in knee OA suggest that combination therapy is more advantageous than single therapy.^26,43^ However, these findings should be interpreted with caution since current evidence relies on a limited number of primary studies.

### Limitations

This study is not without its limitations. We were unable to stratify outcomes by KL grade because the included studies did not report results stratified by KL grade. None of the included studies had an SOC/PBO group at 12 months, which limited our ability to compare the efficacy of injections against a baseline. Finally, heterogeneity is an inherent limitation of NMAs, as variability across included studies introduces bias.^11^ The *P* value does not adjust for the uncertainty or heterogeneity in the network. Injections with wide confidence intervals or high variability may still receive high *P* values.^11^ Given that the current NMA only included 14 trials, we acknowledge that the *P* value should be interpreted with caution, as the *P* value ranking may fluctuate with the addition of future studies.

## Conclusion

This systematic review and NMA evaluated the efficacy of intra-articular injections for reducing hip OA symptoms. We compared 8 types of injections at 3, 6, and 12 months and found no significant differences among all injection types at any time point. There is a lack of placebo-controlled trials with longer-term follow-up, which reflects a gap in the literature. Further research on biologic treatments and combination therapies is needed to clarify their comparative efficacy for hip OA.

## Figures and Tables

**Appendix Figure A1.**
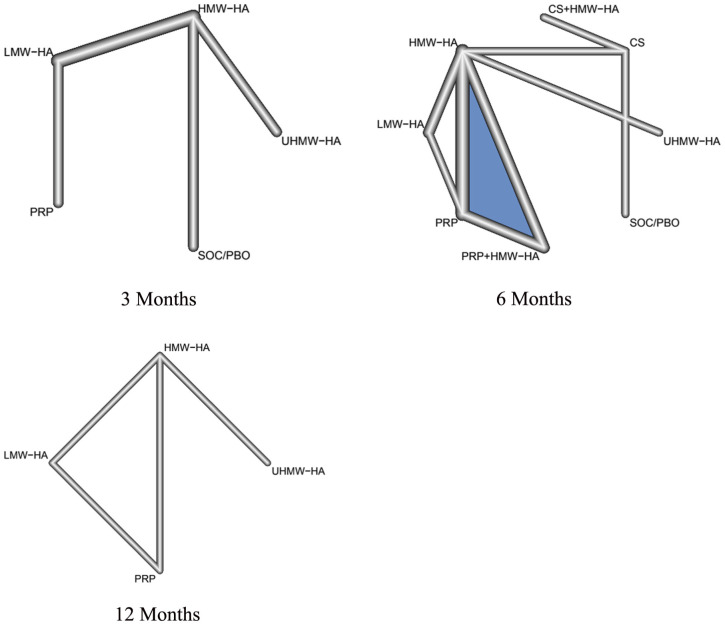
Western Ontario and McMaster Universities Osteoarthritis Index network diagrams at 3 months, 6 months, and 12 months. CS, corticosteroids; HA, hyaluronic acid; HMW, high molecular weight; LMW, low molecular weight; PRP, platelet-rich plasma; SOC/PBO, standard of care/placebo; UHMW, ultra–high molecular weight.

**Appendix Figure A2.**
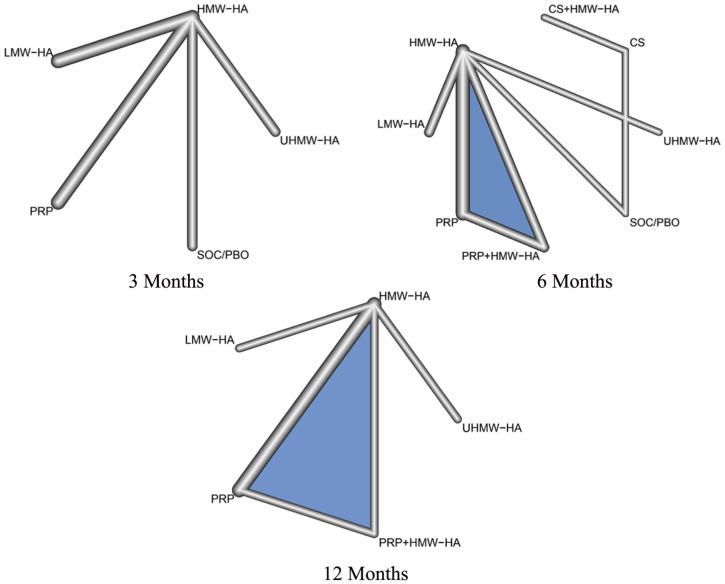
Visual analog scale pain network diagrams at 3 months, 6 months, and 12 months. CS, corticosteroids; HA, hyaluronic acid; HMW, high molecular weight; LMW, low molecular weight; PRP, platelet-rich plasma; SOC/PBO, standard of care/placebo; UHMW, ultra–high molecular weight.

## Footnotes

** References 10, 13, 27, 33, 35, 38, 41, 44, 48.

†† References 4, 6, 13, 32, 33, 35, 38, 39, 44.

## Appendix

**Appendix Table A1:** PRISMA-NMA Checklist*^*a*^*

Section/Topic	Item #	Checklist Item	Location
**TITLE**			
Title	1	Identify the report as a systematic review incorporating an NMA (or related form of meta-analysis)	1
**ABSTRACT**			
Structured summary	2	Provide a structured summary including, as applicable: **B****ackground:** main objectives **M****ethods:** data sources; study eligibility criteria, participants, and interventions; study appraisal; and synthesis methods, such as NMA **R****esults:** number of studies and participants identified; summary estimates with corresponding confidence/credible intervals; treatment rankings may also be discussed; authors may choose to summarize pairwise comparisons against a chosen treatment included in their analyses for brevity **D****iscussion/Conclusion:** limitations; conclusions and implications of findings **O****ther:** primary source of funding; systematic review registration number with registry name	1,2
**INTRODUCTION**			
Rationale	3	Describe the rationale for the review in the context of what is already known, including mention of why an NMA has been conducted	3,4
Objectives	4	Provide an explicit statement of questions being addressed, with reference to participants, interventions, comparisons, outcomes, and study design (PICOS)	3,4
**METHODS**			
Protocol and registration	5	Indicate whether a review protocol exists and if and where it can be accessed (eg, Web address); and, if available, provide registration information, including registration number	5
Eligibility criteria	6	Specify study characteristics (eg, PICOS, length of follow-up) and report characteristics (eg, years considered, language, publication status) used as criteria for eligibility, giving rationale; clearly describe eligible treatments included in the treatment network, and note whether any have been clustered or merged into the same node (with justification)	5
Information sources	7	Describe all information sources (eg, databases with dates of coverage, contact with study authors to identify additional studies) in the search and date last searched	5
Search	8	Present full electronic search strategy for ≥1 database, including any limits used, such that it could be repeated	Table A2-A5
Study selection	9	State the process for selecting studies (ie, screening, eligibility, included in systematic review, and, if applicable, included in the meta-analysis)	5,6
Data collection process	10	Describe method of data extraction from reports (eg, piloted forms, independently, in duplicate) and any processes for obtaining and confirming data from investigators	6
Data items	11	List and define all variables for which data were sought (eg, PICOS, funding sources) and any assumptions and simplifications made	6
Geometry of the network	S1	Describe methods used to explore the geometry of the treatment network under study and potential biases related to it; this should include how the evidence base has been graphically summarized for presentation, and what characteristics were compiled and used to describe the evidence base to readers	6,7
Risk of bias within individual studies	12	Describe methods used for assessing risk of bias of individual studies (including specification of whether this was done at the study or outcome level), and how this information is to be used in any data synthesis	5
Summary measures	13	State the principal summary measures (eg, risk ratio, difference in means); also describe the use of additional summary measures assessed, such as treatment rankings and surface under the cumulative ranking curve values, as well as modified approaches used to present summary findings from meta-analyses	6,7
Planned methods of analysis	14	Describe the methods of handling data and combining results of studies for each NMA; this should include, but not be limited to • Handling of multiarm trials; • Selection of variance structure; • Selection of prior distributions in Bayesian analyses; and • Assessment of model fit	6,7
Assessment of inconsistency	S2	Describe the statistical methods used to evaluate the agreement of direct and indirect evidence in the treatment network(s) studied; describe efforts taken to address its presence when found	7
Risk of bias across studies	15	Specify any assessment of risk of bias that may affect the cumulative evidence (eg, publication bias, selective reporting within studies)	N/A
Additional analyses	16	Describe methods of additional analyses if done, indicating which were prespecified; this may include, but not be limited to, the following: • Sensitivity or subgroup analyses; • Metaregression analyses; • Alternative formulations of the treatment network; and • Use of alternative prior distributions for Bayesian analyses (if applicable)	N/A
**RESULTS**			
Study selection	17	Give numbers of studies screened, assessed for eligibility, and included in the review, with reasons for exclusions at each stage, ideally with a flow diagram	8
Presentation of network structure	S3	Provide a network graph of the included studies to enable visualization of the geometry of the treatment network	Figure A1,A2
Summary of network geometry	S4	Provide a brief overview of characteristics of the treatment network; this may include commentary on the abundance of trials and randomized patients for the different interventions and pairwise comparisons in the network, gaps of evidence in the treatment network, and potential biases reflected by the network structure	15,17
Study characteristics	18	For each study, present characteristics for which data were extracted (eg, study size, PICOS, follow-up period) and provide the citations	Table 1
Risk of bias within studies	19	Present data on risk of bias of each study and, if available, any outcome level assessment	Figure 2
Results of individual studies	20	For all outcomes considered (benefits or harms), present, for each study, 1) simple summary data for each intervention group and 2) effect estimates and confidence intervals. Modified approaches may be needed to deal with information from larger networks	N/A
Synthesis of results	21	Present results of each meta-analysis done, including confidence/credible intervals; in larger networks, authors may focus on comparisons versus a particular comparator (eg, placebo or standard care), with full findings presented in an appendix; league tables and forest plots may be considered to summarize pairwise comparisons; if additional summary measures were explored (such as treatment rankings), these should also be presented	15-18
Exploration for inconsistency	S5	Describe results from investigations of inconsistency. This may include such information as measures of model fit to compare consistency and inconsistency models, *P* values from statistical tests, or summary of inconsistency estimates from different parts of the treatment network	15-18
Risk of bias across studies	22	Present results of any assessment of risk of bias across studies for the evidence base being studied	N/A
Results of additional analyses	23	Give results of additional analyses, if done (eg, sensitivity or subgroup analyses, metaregression analyses, alternative network geometries studied, alternative choice of prior distributions for Bayesian analyses, and so forth)	N/A
**DISCUSSION**			
Summary of evidence	24	Summarize the main findings, including the strength of evidence for each main outcome; consider their relevance to key groups (eg, health care providers, users, and policy makers)	21
Limitations	25	Discuss limitations at study and outcome level (eg, risk of bias), and at review level (eg, incomplete retrieval of identified research, reporting bias); comment on the validity of the assumptions, such as transitivity and consistency; comment on any concerns regarding network geometry (eg, avoidance of certain comparisons)	23
Conclusion	26	Provide a general interpretation of the results in the context of other evidence, and implications for future research	23,24
**FUNDING**			
Funding	27	Describe sources of funding for the systematic review and other support (eg, supply of data); role of funders for the systematic review; this should also include information regarding whether funding has been received from manufacturers of treatments in the network and/or whether some of the authors are content experts with professional conflicts of interest that could affect use of treatments in the network	N/A

a NMA, network meta-analysis; PRISMA, Preferred Reporting Items for Systematic Reviews and Meta-Analyses.

**Appendix Table A2:** Web of Science (Core Collection) Search Strategy

Line #	Search statement	Results
1	TS=(platelet-rich OR platelet-derived OR (platelet NEAR/3 concentrate) OR (platelet NEAR/3 plasma) OR (platelet NEAR/3 gel) OR (platelet NEAR/3 fibrin) OR (platelet NEAR/3 growth factor) OR (platelet NEAR/3 lysate) OR "PRP" OR "PRGF" OR "PRF" OR (autologous NEAR/3 plasma) OR (autologous NEAR/3 protein) OR "APS" OR "ACP")	115516
2	TS=(steroid* OR cortico* OR glucocortico* OR dexamethason* OR methylprednisolon* OR betamethason* OR triamcinolon* OR hydroxycortico* OR hydrocort* OR cortison* OR prednison* OR prednisolon*)	719476
3	TS=(orthobiolog* OR prolotherap* OR (proliferat* NEAR/5 therap*) OR (proliferat* NEAR/5 inject*) OR (regenerat* NEAR/5 therap*) OR (regenerat* NEAR/5 inject*) OR (biolog* NEAR/5 therap*) OR (biolog* NEAR/5 inject*) OR "MSC" OR "MSCs" OR allogen* OR stem cell* OR mesenchymal OR stromal OR progenitor OR (marrow NEAR/3 concentrat*) OR (marrow NEAR/3 aspirat*) OR "BMAC" OR (adipo* NEAR/3 microfrag*) OR (adipo NEAR/3 micro-frag*) OR lipoaspirat* OR “SVF” OR “ASA” OR “placent*” OR umbilic* OR amnio*)	1279381
4	TS= (hyaluron* OR hylan* OR viscosup* OR synvisc* OR orthovisc* OR monovisc* OR durolane)	62105
5	#1 OR #2 OR #3 OR #4	2108092
6	TS= (intra-articular OR intraarticular OR inject*)	1138945
7	TS=(osteoarthr* OR arthr* OR degenerat*)	969483
8	TS=(hip)	230197
9	#7 AND #8	88397
10	TS=(random*)	2458213
11	#5 AND #6 AND #9 AND #10	526

**Appendix Table A3:** Ovid Embase Search Strategy

Line #	Search statement	Results
1	(platelet-rich or platelet-derived or (platelet adj3 (concentrate or plasma or gel or fibrin or growth factor or lysate)) or "PRP" or "PRGF" or "PRF" or (autologous adj3 (plasma or protein)) or "APS" or "ACP").ti,ab.	125772
2	exp thrombocyte rich plasma/ or exp platelet-rich fibrin/	20184
3	(steroid* or cortico* or glucocortico* or dexamethason* or methylprednisolon* or betamethason* or triamcinolon* or hydroxycortico* or hydrocort* or cortison* or prednison* or prednisolon*).ti,ab.	968376
4	exp steroid/ or exp fluorinated steroid/ or exp corticosteroid/ or exp corticosteroid therapy/ or exp glucocorticoid/ or exp dexamethasone/ or exp dexamethasone sodium phosphate/ or exp methylprednisolone/ or exp methylprednisolone acetate/ or exp triamcinolone/ or exp triamcinolone acetonide/ or exp betamethasone/ or exp betamethasone sodium phosphate/ or exp betamethasone acetate/ or exp betamethasone acetate plus betamethasone sodium phosphate/ or exp prednisolone/ or exp prednisone/ or exp hydrocortisone/ or exp cortisone/	2006383
5	(orthobiolog* or prolotherap* or ((proliferat* or regenerat* or biolog*) adj5 (therap* or inject*)) or "MSC" or "MSCs" or allogen* or stem cell* or mesenchymal or stromal or progenitor or (marrow adj3 (concentrat* or aspirat*)) or "BMAC" or (adipo* adj3 (microfrag* or micro-frag*)) or lipoaspirat* or "SVF" or "ASA" or placent* or umbilic* or amnio*).ti,ab.	1308857
6	exp biological product/ or exp prolotherapy/ or exp stem cell/ or exp mesenchymal stroma cell/ or exp mesenchymal stem cell/ or exp mesenchymal stromal cell therapy/	1421633
7	(hyaluron* or hylan* or viscosup* or synvisc* or orthovisc* or monovisc* or durolane).ti,ab.	60390
8	exp viscosupplementation/ or exp hyaluronic acid/ or exp hyaluronic acid derivative/	57645
9	or/1-8	4520765
10	(intra-articular or intraarticular or inject*).ti,ab.	1276297
11	exp intraarticular drug administration/ or exp injection/	264629
12	10 or 11	1306431
13	(osteoarthr* or arthr* or degenerat*).ti,ab. and hip.mp.	85685
14	exp hip osteoarthritis/ or exp "Western Ontario and McMaster Universities Osteoarthritis Index"/ or exp "Hip Disability and Osteoarthritis Outcome Score"/	24326
15	13 or 14	95444
16	(Randomized controlled trial/ or Controlled clinical study/ or random$.ti,ab. or randomization/ or intermethod comparison/ or placebo.ti,ab. or (compare or compared or comparison).ti. or ((evaluated or evaluate or evaluating or assessed or assess) and (compare or compared or comparing or comparison)).ab. or (open adj label).ti,ab. or ((double or single or doubly or singly) adj (blind or blinded or blindly)).ti,ab. or double blind procedure/ or parallel group$1.ti,ab. or (crossover or cross over).ti,ab. or ((assign$ or match or matched or allocation) adj5 (alternate or group$1 or intervention$1 or patient$1 or subject$1 or participant$1)).ti,ab. or (assigned or allocated).ti,ab. or (controlled adj7 (study or design or trial)).ti,ab. or (volunteer or volunteers).ti,ab. or human experiment/ or trial.ti.) not (((random$ adj sampl$ adj7 ("cross section$" or questionnaire$1 or survey$ or database$1)).ti,ab. not (comparative study/ or controlled study/ or randomi?ed controlled.ti,ab. or randomly assigned.ti,ab.)) or (Cross-sectional study/ not (randomized controlled trial/ or controlled clinical study/ or controlled study/ or randomi?ed controlled.ti,ab. or control group$1.ti,ab.)) or (((case adj control$) and random$) not randomi?ed controlled).ti,ab. or (Systematic review not (trial or study)).ti. or (nonrandom$ not random$).ti,ab. or "Random field$".ti,ab. or (random cluster adj3 sampl$).ti,ab. or ((review.ab. and review.pt.) not trial.ti.) or ("we searched".ab. and (review.ti. or review.pt.)) or "update review".ab. or (databases adj4 searched).ab. or ((rat or rats or mouse or mice or swine or porcine or murine or sheep or lambs or pigs or piglets or rabbit or rabbits or cat or cats or dog or dogs or cattle or bovine or monkey or monkeys or trout or marmoset$1).ti. and animal experiment/) or (Animal experiment/ not (human experiment/ or human/)))	5717982
17	9 and 12 and 15 and 16	1112

**Appendix Table A4:** Ovid MEDLINE Search Strategy

Line #	Search statement	Results
1	(platelet-rich or platelet-derived or (platelet adj3 (concentrate or plasma or gel or fibrin or growth factor or lysate)) or "PRP" or "PRGF" or "PRF" or (autologous adj3 (plasma or protein)) or "APS" or "ACP").ti,ab.	80139
2	exp Platelet-Rich Plasma/	6846
3	(steroid* or cortico* or glucocortico* or dexamethason* or methylprednisolon* or betamethason* or triamcinolon* or hydroxycortico* or hydrocort* or cortison* or prednison* or prednisolon*).ti,ab.	567564
4	exp Steroids/ or exp Dexamethasone/ or exp Methylprednisolone/ or exp Prednisolone/ or exp Prednisone/ or exp Hydrocortisone/ or exp Cortisone/ or exp Betamethasone/ or exp Triamcinolone/	926577
5	(orthobiolog* or prolotherap* or ((proliferat* or regenerat* or biolog*) adj5 (therap* or inject*)) or "MSC" or "MSCs" or allogen* or stem cell* or mesenchymal or stromal or progenitor or (marrow adj3 (concentrat* or aspirat*)) or "BMAC" or (adipo* adj3 (microfrag* or micro-frag*)) or lipoaspirat* or "SVF" or "ASA" or placent* or umbilic* or amnio*).ti,ab.	784347
6	exp Prolotherapy/ or exp "Cell- and Tissue-Based Therapy"/ or exp Stem Cells/ or exp Biological Products/	1224303
7	(hyaluron* or hylan* or viscosup* or synvisc* or orthovisc* or monovisc* or durolane).ti,ab.	39444
8	exp Hyaluronic Acid/ or exp Viscosupplementation/	26143
9	or/1-8	2969770
10	(intra-articular or inject* or intraarticular).ti,ab.	761111
11	exp Injections/	297667
12	10 or 11	920494
13	(osteoarthr* or arthr* or degenerat*).ti,ab. and hip.mp.	54084
14	exp Osteoarthritis, Hip/	9856
15	13 or 14	55647
16	((randomized controlled trial or controlled clinical trial).pt. or random*.mp. or placebo.ab. or drug therapy.fs. or trial.ab. or groups.ab.) not (exp animals/ not humans.sh.)	4785737
17	9 and 12 and 15 and 16	394

**Appendix Table A5:** Cochrane Central Register of Controlled Trials (CENTRAL) Search Strategy*^*a*^*

Line #	Search statement	Results
#1	(platelet-rich or platelet-derived or (platelet NEAR/3 (concentrate or plasma or gel or fibrin or growth factor or lysate)) or "PRP" or "PRGF" or "PRF" or (autologous NEAR/3 (plasma or protein)) or "APS" or "ACP"):ti,ab	10,120
#2	MeSH descriptor: [Platelet-Rich Plasma] explode all trees	967
#3	(steroid* or cortico* or glucocortico* or dexamethason* or methylprednisolon* or betamethason* or triamcinolon* or hydroxycortico* or hydrocort* or cortison* or prednison* or prednisolon*):ti,ab	81,282
#4	MeSH descriptor: [Steroids] explode all trees	68,923
#5	MeSH descriptor: [Dexamethasone] explode all trees	5687
#6	MeSH descriptor: [Methylprednisolone] explode all trees	3109
#7	MeSH descriptor: [Prednisolone] explode all trees	5586
#8	MeSH descriptor: [Prednisone] explode all trees	4497
#9	MeSH descriptor: [Hydrocortisone] explode all trees	6812
#10	MeSH descriptor: [Cortisone] explode all trees	178
#11	MeSH descriptor: [Betamethasone] explode all trees	1655
#12	MeSH descriptor: [Triamcinolone] explode all trees	1648
#13	(orthobiolog* or prolotherap* or ((proliferat* or regenerat* or biolog*) NEAR/5 (therap* or inject*)) or "MSC" or "MSCs" or allogen* or stem cell* or mesenchymal or stromal or progenitor or (marrow NEAR/3 (concentrat* or aspirat*)) or "BMAC" or (adipo* NEAR/3 (microfrag* or micro-frag*)) or lipoaspirat* or "SVF" or "ASA" or placent* or umbilic* or amnio*):ti,ab	68,957
#14	MeSH descriptor: [Prolotherapy] explode all trees	42
#15	MeSH descriptor: [Cell- and Tissue-Based Therapy] explode all trees	8748
#16	MeSH descriptor: [Stem Cells] explode all trees	1152
#17	MeSH descriptor: [Biological Products] explode all trees	34,103
#18	(hyaluron* or hylan* or viscosup* or synvisc* or orthovisc* or monovisc* or durolane):ti,ab	5451
#19	MeSH descriptor: [Hyaluronic Acid] explode all trees	2139
#20	MeSH descriptor: [Viscosupplementation] explode all trees	62
#21	#1 or #2 or #3 or #4 or #5 or #6 or #7 or #8 or #9 or #10 or #11 or #12 or #13 or #14 or #15 or #16 or #17 or #18 or #19 or #20	238,487
#22	(intra-articular or inject* or intraarticular):ti,ab	109,409
#23	MeSH descriptor: [Injections] explode all trees	25,077
#24	#22 or #23	121,597
#25	(osteoarthr* or arthr* or degenerat*):ti,ab	68,198
#26	hip:ti,ab,kw	29,415
#27	#25 and #26	8684
#28	MeSH descriptor: [Osteoarthritis, Hip] explode all trees	1235
#29	#27 or #28	8833
#30	#21 and #24 and #29	407
#31	#30 in Trials	402

a MeSH, Medical Subject Headings.
